# Compartmentalization of the Inflammatory Response in the Pericardial Cavity in Patients Undergoing Cardiac Surgery

**DOI:** 10.3390/ijms252413720

**Published:** 2024-12-23

**Authors:** Mohammad M. El-Diasty, Javier Rodríguez, Luis Pérez, Souhayla Souaf, Sonia Eiras, Angel L. Fernández

**Affiliations:** 1 Cardiac Surgery Department, Harrington Heart and Vascular Institute, University Hospitals Cleveland Medical Center, Cleveland, OH 44106, USA; 2 Division of Clinical Biochemistry, University Hospital, 15706 Santiago de Compostela, Spain; 3 Division of Cardiac Surgery, University Hospital, 15706 Santiago de Compostela, Spain; 4 Laboratory of Cardiovascular Research, University Hospital, 15706 Santiago de Compostela, Spain; 5 Division of Cardiac Surgery, University Hospital, Department of Surgery, University of Santiago de Compostela, 15706 Santiago de Compostela, Spain

**Keywords:** cardiopulmonary bypass, cardiac surgery, inflammatory response, pericardial fluid, pericardium, cytokines, inflammation, interleukins, interleukin 1α (IL-1α), interleukin 1β (IL-1β), interleukin 2 (IL-2), interleukin 4 (IL-4), interleukin 6 (IL-6), interleukin 8 (IL-8), interleukin 10 (IL-10), tumor necrosis factor-α (TNF-α), interferon-γ (IFN-γ), growth factors, adhesion molecules

## Abstract

The systemic inflammatory response after cardiopulmonary bypass has been widely studied. However, there is a paucity of studies that focus on the local inflammatory changes that occur in the pericardial cavity. The purpose of this study is to assess the inflammatory mediators in the pericardial fluid of patients undergoing cardiac surgery. We conducted a prospective cohort study on patients undergoing aortic valve replacement. Pericardial fluid and peripheral venous blood samples were collected after the opening of the pericardium. Additional samples were obtained from peripheral blood and the pericardial fluid shed through mediastinal drains 24 and 48 h after surgery. Levels of interleukin 1α (IL-1α), interleukin 1β (IL-1β), interleukin 2 (IL-2), interleukin 4 (IL-4), interleukin 6 (IL-6), interleukin 8 (IL-8), interleukin 10 (IL-10), tumor necrosis factor-α (TNF-α), interferon-γ (IFN-γ), vascular endothelial growth factor (VEGF), monocyte chemotactic protein-1 (MCP-1), epidermal growth factor (EGF), soluble E-selectin, L-selectin, P-selectin, intercellular adhesion molecule-1 (ICAM-1), and vascular cell adhesion molecule-1 (VCAM-1) were determined in all pericardial fluid and serum samples. A total of 45 patients with a mean age of 74 years were included, of which 66% were males. Serum levels of IL-6, IL-8, and MCP-1 were significantly increased at 24 and 48 h after surgery. No significant changes were observed in the serum levels of the remaining mediators. A significant increase of postoperative pericardial fluid levels of IL-1α, IL-1β, IL-6, IL-8, IL-10, IFN-γ, VEGF, MCP-1, VCAM-1, and P-selectin was observed at 24 and 48 h after surgery. There is a robust systemic and pericardial inflammatory response after cardiac surgery on cardiopulmonary bypass. However, postoperative pericardial inflammatory activity shows a distinct pattern and is more marked than at the systemic level. These findings suggest that there is a compartmentalization of the inflammatory response within the pericardial cavity after cardiac surgery.

## 1. Introduction

Cardiac surgery is associated with a systemic inflammatory response that plays a major role in the development of postoperative organ dysfunction and adverse clinical outcomes [1,2,3]. Postoperative inflammatory response can be triggered by the contact of blood with the surface of the cardiopulmonary bypass circuit, hypoperfusion of body organs, and intraoperative mechanical tissue trauma [4,5]. Important features of postoperative inflammatory reactions include the following: (1) the activation of the leukocytes, endothelial cells, platelets, coagulation, and complement and fibrinolytic systems; (2) the increase in circulating levels of proinflammatory mediators; (3) the alterations in the metabolism of nitric oxide; and (4) the increase in the production of oxygen free radicals [4,6].

Numerous studies have identified different mediators and have characterized the postoperative systemic inflammatory reaction in cardiac surgery at the systemic level [7,8,9,10,11,12,13,14,15]. In contrast, there are hardly any studies that focus on postoperative inflammatory activity in the pericardial compartment. It is, however, well established that the pericardial space plays a role in the synthesis and accumulation of a wide variety of inflammatory mediators under basal conditions [14,16,17,18,19,20] as well as in different pathological disorders such as purulent [21], viral [19], rheumatic [22], autoimmune [19], and tuberculous pericarditis [23], as well as in congestive heart failure [24].

The objective of this study is to determine the patterns of changes in postoperative levels of inflammatory mediators in blood and pericardial fluid in patients undergoing elective cardiac surgery with cardiopulmonary bypass.

## 2. Results

Forty-five consecutive patients who fulfilled the inclusion criteria were included in this study. The mean age was 74.5 ± 6.1 years. Table 1 summarizes the clinical data of the included patients.

It was found that there was a statistically significant increase in the postoperative serum levels of IL-6 and IL-8. No significant changes were observed regarding the other inflammatory mediators.

Table 2 summarizes the basal, 24 h, and 48 h postoperative values of cytokines and growth factors in serum. Table 3 summarizes the basal, 24 h, and 48 h postoperative values of soluble adhesion molecules in serum.

It was found that there was a statistically significant increase in the postoperative pericardial levels of IL-1α, IL-1β, IL-6, IL-8, IL-10, IFN-γ, VEGF, MCP-1, VCAM-1, and P-selectin. No significant changes were observed regarding the other inflammatory mediators.

Table 4 summarizes the basal, 24 h, and 48 h postoperative values of cytokines and growth factors in the pericardial fluid. Table 5 summarizes the basal, 24 h, and 48 h postoperative values of soluble adhesion molecules in the pericardial fluid.

## 3. Discussion

Our results demonstrate that there is a clear inflammatory response at the systemic and local pericardial levels during the first 48 h postoperatively in patients undergoing cardiac surgery on cardiopulmonary bypass. However, the accumulation of inflammatory mediators observed in the pericardial fluid is more intense than in the serum.

Cardiac surgery with extracorporeal circulation can provoke a systemic inflammatory response that can result in adverse clinical events such as acute lung injury, hemodynamic instability, coagulation disorders, acute kidney injury, and neurocognitive disorders [1,2,5]. This systemic reaction is associated with a surge in serum levels of several inflammatory mediators that typically peak within the first 24–48 h postoperatively and includes IL-1β [9], IL-5 [9], IL-6 [6,7,8,9,10,11,12,20], IL-8 [9,10], IL-10 [6,9,10,12,15], IL-13 [9,13], TNF-α [6,7,8,9], VEGF [12,15], and MCP-1 [10,11,12,15,20].

However, postoperative changes in serum levels of some other inflammatory mediators showed less consistent results. While some studies showed increased postoperative serum levels of IL-2 [9], IL-4 [13], and IFN-γ [9,13], others showed reductions in these levels [10,12].

In our study we were able to observe a systemic response with a significant increase in serum levels of IL-6, IL-8, and MCP-1 without significant changes in the rest of the mediators.

Regarding the increase in the levels of inflammatory mediators in pericardial fluid in the postoperative period, this has been suggested based on the observation of elevated levels of IL-1β, IL-6, IL-10, and TNF-α in the mediastinal shed blood used for autotransfusion after cardiac surgery [25,26,27,28]. However, there is a paucity of studies regarding the composition of the pericardial exudate after open heart surgery despite the various reports that showed significant postoperative increases of pericardial IL-5 [29], IL-6 [14,20,30], IL-8, IL-10, and MCP-1 [20], as well as TNF-α [30]. In the present study we were able to demonstrate a significant increase in the levels of postoperative pericardial fluid of IL-1α, IL-1β, IL-6, IL-8, IL-10, IFN-γ, VEGF, MCP-1, VCAM-1, and P-selectin. This acute local reaction at the pericardial level was characterized by greater amplitude and intensity than that observed at the systemic level, suggesting the presence of compartmentalization of the acute postoperative inflammatory reaction within the pericardial space.

Factors that have been suggested to trigger the pericardial inflammatory reaction and the consequent increase in pericardial inflammatory mediators include the following: (1) direct tissue trauma from surgical manipulation of the pericardium and heart; (2) myocardial injury from ischemia–reperfusion; and (3) the presence of retained blood and platelet activation in the pericardial sac in the early postoperative period. It has also been reported that epicardial adipose tissue, cardiomyocytes, stromal cells, pericardial mesothelium and inflammatory cells present in the pericardial fluid (such as monocytes, macrophages, and T lymphocytes) can produce IL-6, IL-10, VEGF, INF-γ, and MCP-1 [31,32,33,34].

It has also been reported that in the postoperative period a profound modification of the cellular population of the pericardial fluid occurs. Under basal conditions, there is a predominance of macrophages, monocytes, mesothelial cells, and lymphocytes, while neutrophils are typically absent [16,20,29,34,35]. However, as early as four hours postoperatively, there is a prevalence of neutrophils, which peaks around 24 h [20,29,35]. These neutrophils play a role in the synthesis of IL-1α, IL-1β, IL-4, IL-6, TNF-α, and VEGF [36], thus contributing to the marked local increase of these mediators in the postoperative period.

Our study showed that the intense postoperative inflammatory reaction within the pericardial compartment is distinct and does not replicate the same pattern of the systemic response as evidenced by the discrepancy in the levels of pericardial and serum mediators. In this regard, it should be highlighted that most of the strategies used to attenuate the inflammatory reaction after cardiopulmonary bypass (such as the use of steroids, heparin-coated circuits, leukocytic filters, and cytokine hemadsorption systems) have been shown to reduce the levels of inflammatory mediators in blood [37] but do not seem to have demonstrated significant clinical efficacy in the prevention of postoperative complications [38]. In addition, these strategies have not been designed to target the pericardial component of the postoperative inflammatory response.

The use of off-pump coronary surgical techniques has largely been thought to reduce the systemic inflammatory response that may be triggered by the use of the cardiopulmonary bypass machine. Randomized controlled trials comparing the impact of off-pump and on-pump coronary surgery on systemic inflammatory response have demonstrated that off-pump surgery can significantly attenuate the systemic inflammatory response but does not completely suppress it. Regarding postoperative IL-6 and IL-8 values, the data are inconclusive. While some authors have observed lower plasma levels of IL-6 and IL-8 in patients undergoing off-pump surgery [10], others were unable to demonstrate these differences [12,39]. In fact, other studies have shown lower production of IL-8 but not IL-6 in off-pump surgery [40,41]. These mixed results may be explained by the fact that the release of inflammatory mediators during off-pump surgery may be triggered by direct surgical trauma, manipulation of the heart, and the administration of certain drugs such as protamine and anesthetic agents.

The relationship between the pericardial inflammatory response and local complications (such as postoperative atrial fibrillation, postoperative mediastinal adhesions, post-cardiotomy syndrome, and constrictive pericarditis) has been suggested by several authors [20,29,30,42,43]. For instance, it was demonstrated that elevated mediators of pericardial inflammation were associated with the development of postoperative atrial fibrillation [14,44]. It is therefore reasonable to suggest that the development of specific measures to attenuate the local pericardial inflammatory reaction may help reduce these postoperative complications [38].

The main limitation of this study is the wide variability in the intensity of the inflammatory response to cardiopulmonary bypass between patients. This may be explained by the heterogeneity in baseline patients’ clinical characteristics, such as age, sex, cardiovascular risk factors, transfusions received during the intervention, as well as genetic factors [5,8,45]. Also, we were unable to study the inflammatory response in the cardio-pericardial compartment beyond the early phase of the postoperative period, since in most patients the mediastinal drains were removed after 48 h, therefore limiting access to pericardial fluid samples.

## 4. Materials and Methods

This is a prospective cohort study that includes adult patients with aortic stenosis who are candidates for elective isolated aortic valve replacement surgery. Exclusion criteria included the following: history of pericardial disease, previous cardiac intervention, presence of coronary heart disease, diabetes mellitus, end-stage renal dysfunction on renal replacement therapy, history of inflammatory or autoimmune conditions, atrial fibrillation, infective endocarditis, preoperative insertion of intra-aortic balloon pump of counter-pulsation, intraoperative opening of the pleural cavity, early reintervention due to postoperative bleeding/tamponade, electrocardiographic, and/or echocardiographic findings suggestive of postoperative acute myocardial infarction, and recent use of anti-inflammatory agents.

The study protocol was compliant with the ethical guidelines of the 1975 Declaration of Helsinki, and it was approved by the Institutional Ethics Committee. A written informed consent was obtained from all patients. All surgical procedures were performed via median sternotomy. Before giving heparin, the pericardial sac was incised vertically, and a 5–10 mL sample of pericardial fluid was carefully withdrawn. At the same time, a peripheral venous sample was also extracted. Both samples were transferred to sterile tubes that were kept on ice while being transferred immediately to the laboratory. All patients were operated on under cardiopulmonary bypass and moderate systemic hypothermia (32–34 °C). Once the aorta was clamped cold (6 °C), hyperkalemic blood cardioplegia solution was administered through the aortic root, achieving adequate cardiac arrest. For maintenance, subsequent doses of cardioplegia were administered every 20 min at 6 °C. In order to attenuate ischemia–reperfusion injury and to reduce the inflammatory response, a single dose of warm (37 °C) reperfusion cardioplegic solution, “Hot Shot”, was administered just before reperfusion. At the end of the operation, before the wound closure, two mediastinal drains were inserted into the pericardial cavity. Four more samples were obtained, i.e., two from peripheral blood and two from the pericardial fluid shed through mediastinal drains at 24 and 48 h after surgery.

After separating the cellular components by centrifugation at 3500 rpm for 10 min at 4 °C, the supernatant fluid was isolated and stored at −30 °C. Interleukins, growth factors, and adhesion molecules were simultaneously measured in serum and pericardial fluid using the Evidence^®^ biochip array technology (Randox Laboratories Ltd., Crumlin, Antrim, UK). This biochip array technology uses the sandwich chemiluminescent immunoassay to detect levels of multiple analytes at the same time from a single sample. Two different multi-analyte panels were used in this study, i.e., the Evidence^®^ Cytokine and Growth Factors array. These were used for simultaneous quantitative detection of interleukins IL-1α, IL-1β, IL-2, IL-4, IL-6, IL-8, and IL-10, tumor necrosis factor-α (TNF-α), interferon-γ (IFN-γ), vascular endothelial growth factor (VEGF), monocyte chemotactic protein-1 (MCP-1), and epidermal growth factor (EGF). The Evidence^®^ Adhesion Molecules array was used for the quantitative detection of soluble E-selectin, L-selectin, P-selectin, intercellular adhesion molecule-1 (ICAM-1), and vascular cell adhesion molecule-1 (VCAM-1).

Samples were processed using the automated biochip array analyzer according to the manufacturer’s instructions as previously described [16,46].

A minimum of 500 µL of sample is required for standard sample cups of the Cytokine and Growth Factors array. Reagent composition includes the following: Cytokine Assay Diluent: 20 mM Tris-buffered saline pH 7.2 containing a protein matrix, detergent, and preservatives; Cytokine Conjugate: 20 mM Tris-buffered saline pH 7.5 containing a protein matrix, detergent, preservatives, and assay-specific antibodies labeled with horseradish peroxidase (HRP); and Cytokine Biochips: Solid-phase substrate containing an immobilized antibody in discrete test regions. Instrument calibration is performed using the Randox Cytokine calibrators (Randox Laboratories Ltd., Crumlin, Antrim, UK). Calibration is performed upon initial setup of the system. Intermittent calibration of the system is also performed to ensure accurate and reliable results. A multi-point calibration is conducted with the change in reagent lot or as indicated by quality control procedures. Results are processed automatically using dedicated software 2.0.0.

Samples are diluted 1 in 10 using working strength wash buffer (50 µL of sample added to 450 µL of wash buffer and mixed thoroughly) for the determination of adhesion molecules. Minimal volume required is 25 µL of serum. Reagent array composition includes the following: Adhesion Molecules Assay Diluent: 19 mM Tris-buffered saline, pH 7.2, containing a protein matrix, surfactant, and preservatives; Adhesion Molecules Conjugate: 19 mM Tris-based buffer, pH 7.5, containing a protein matrix, surfactant, preservatives, and assay-specific antibodies labeled with horseradish peroxidase (HRP); and Adhesion Molecules Biochips: Solid-phase substrate containing discrete test regions of an immobilized antibody. A nine-point calibration is performed using the Adhesion Molecules Evidence^®^ calibrators (Randox Laboratories Ltd., Crumlin, Antrim, UK). Calibration is also performed upon initial setup of the system. Intermittent calibration of the system is necessary to ensure accurate and reliable results. A multi-point calibration is performed with the change in reagent lot or as indicated by quality control procedures. Results are processed automatically using dedicated software 2.0.0. However, patient sample results must be manually multiplied by 10 to account for sample dilution.

### Statistical Analysis

Categorical variables were expressed as numbers and percentages. Continuous variables were presented as means ± standard deviation (SD) and were compared by using a two-tailed Student’s t test or Mann–Whitney test. A *p*-value of <0.05 was considered statistically significant. The software SigmaStat 3.1 was used (Systat Software, Inc., San Jose, CA, USA).

## 5. Conclusions

In patients undergoing cardiac surgery on cardiopulmonary bypass, the postoperative inflammatory reaction in the pericardial compartment is more intense than that observed at the systemic level. Effective therapeutic strategies to reduce postoperative inflammatory response in this group of patients should be designed to specifically target this pericardial component for maximal clinical efficacy.

## Figures and Tables

**Table 1:** Demographic and preoperative characteristics of patients.

Age (years)	74.5 ± 6.1
Sex female/male (*n*)	15/30
BMI (kg/m^2^)	28.7 ± 3.1
Arterial hypertension (%)	42.2
LVEF (%)	54 ± 7
Total protein (g/dL)	6.4 ± 0.6
Albumin (g/dL)	4.2 ± 0.3
Creatinine (mg/dL)	1 ± 0.2
Hemoglobin (g/dL)	13.5 ± 1.1
Aortic cross-clamp time (min)	64.2 ± 8.5
CPB time (min)	75.8 ± 15.9

BMI: body mass index (weight kg/height m^2^); LVEF: left ventricular ejection fraction; CPB: cardiopulmonary bypass.

**Table 2:** Basal and postoperative serum levels of cytokines and growth factors.

	Basal	24 h Postop	48 h Postop	*p*-Value
IL-1α	0.12 ± 0.21	0.52 ± 0.23	0.41 ± 0.66	0.497
IL-1β	0.29 ± 0.15	0.34 ± 0.46	0.87 ± 0.52	0.613
IL-2	2.71 ± 3.14	1.69 ± 2.45	2.6 ± 2.88	0.781
IL-4	6.78 ± 2.55	6.36 ± 3.52	5.97 ± 2.31	0.394
IL-6	11.49 ± 6.08	201 ± 33.6	279 ± 65.45	<0.001
IL-8	7.82 ± 5.69	38.26 ± 9.33	430 ± 24.5	<0.001
IL-10	0.25 ± 0.24	1.77 ± 0.31	2.01 ± 0.72	0.418
TNF-α	3.18 ± 1.06	4.11 ± 1.03	4.03 ± 1.22	0.529
IFN-γ	0.83 ± 0.65	0.18 ± 0.31	1.61 ± 0.53	0.682
VEGF	163.3 ± 129.2	191 ± 72.8	205 ± 103.6	0.297
MCP-1	301.6 ± 58.32	556 ± 80.12	514 ± 94.2	<0.05
EGF	81.57 ± 40.78	49.92 ± 28.76	32.11 ± 25.5	0.392

IL-1α: interleukin 1α; IL-1β: interleukin 1β; IL-2: interleukin 2; IL-4: interleukin 4; IL-6: interleukin 6; IL-8: interleukin 8; IL-10: interleukin 10, TNF-α: tumor necrosis factor-α; IFN-γ: interferon-γ; VEGF: vascular endothelial growth factor; MCP-1: monocyte chemotactic protein-1; EGF: epidermal growth factor (EGF).

**Table 3:** Basal and postoperative serum levels of soluble adhesion molecules.

	Basal	24 h Postop	48 h Postop	*p*-Value
VCAM-1 (ng/mL)	1245.5 ± 301.2	1769 ± 328	1502 ± 385	0.484
ICAM-1 (ng/mL)	568.4 ± 137.7	862.3 ± 149	707.9 ± 116	0.371
E-selectin (ng/mL)	17.44 ± 3.82	21.03 ± 5.9	18.6 ± 4.67	0.392
P-selectin (ng/mL)	272.26 ± 51.3	259 ± 38.2	242 ± 49.6	0.520
L-selectin (ng/mL)	1486.92 ± 208.5	1401 ± 197.4	1352.3 ± 236	0.649

ICAM-1: intercellular adhesion molecule-1; VCAM-1: vascular cell adhesion molecule-1.

**Table 4:** Basal and postoperative pericardial fluid levels of cytokines and growth factors.

	Basal	24 h Postop	48 h Postop	*p*-Value
IL-1α	0.140 ± 0.29	12.36 ± 2.75	10.9 ± 2.87	<0.001
IL-1β	0.48 ± 0.6	36.89 ± 6.13	25.45 ± 5.76	<0.001
IL-2	8.23 ± 11.45	3.93 ± 2.01	6.16 ± 3.77	0.503
IL-4	5.85 ± 4.82	5.02 ± 2.36	7.34 ± 3.91	0.648
IL-6	183.81 ± 94.49	2056.2 ± 103.4	1755.01 ± 124.8	<0.001
IL-8	28.12 ± 20.92	3871.5 ± 245.9	3640 ± 194.6	<0.001
IL-10	0.52 ± 0.41	40.83 ± 11.67	26.05 ± 9.98	<0.001
TNF-α	4.271 ± 0.28	8.15 ± 3.62	7.11 ± 5.29	0.251
IFN-γ	5.34 ± 5.12	18.6 ± 5.79	21.02 ± 6.48	<0.001
VEGF	22.8 ± 16.36	902.14 ± 118.9	1250.6 ± 190.5	<0.001
MCP-1	1206.98 ± 285.49	3394.2 ± 392.3	2458.04 ± 335	<0.01
EGF	2.97 ± 1.67	19.23 ± 22.25	24.84 ± 30.1	0.385

IL-1α: interleukin 1α; IL-1β: interleukin 1β; IL-2: interleukin 2; IL-4: interleukin 4; IL-6: interleukin 6; IL-8: interleukin 8; IL-10: interleukin 10, TNF-α: tumor necrosis factor-α; IFN-γ: interferon-γ; VEGF: vascular endothelial growth factor; MCP-1: monocyte chemotactic protein-1; EGF: epidermal growth factor (EGF).

**Table 5:** Basal and postoperative pericardial fluid levels of soluble adhesion molecules.

	Basal	24 h Postop	48 h Postop	*p*-Value
VCAM-1	218.3 ± 148.3	923.4 ± 225	815.5 ± 193.7	<0.001
ICAM-1	301.7 ± 75.1	501.23 ± 96	584.1 ± 170.6	0.817
E-selectin	3.8 ± 1.75	11.05 ± 4.12	12.6 ± 5.49	0.452
P-selectin	19.35 ± 2.6	307.82 ± 30.9	256.13 ± 22.3	<0.001
L-selectin	695.1 ± 177.3	940.92 ± 189.3	1172 ± 204.3	0.208

ICAM-1: intercellular adhesion molecule-1; VCAM-1: vascular cell adhesion molecule-1.

## Data Availability

Data are available upon reasonable request to the corresponding author. The data are not publicly available due to ethical restrictions.
